# Identification of potential micro-messenger RNAs (miRNA–mRNA) interaction network of osteosarcoma

**DOI:** 10.1080/21655979.2021.1947065

**Published:** 2021-07-12

**Authors:** Keteng Xu, Pei Zhang, Jiale Zhang, Huahong Quan, Jingcheng Wang, Yuan Liang

**Affiliations:** a Department of Orthopedics, The Second Xiangya Hospital of Central South University, Changsha, China; b Department of Orthopedics, Clinical Medical College, Yangzhou University, Northern Jiangsu People's Hospital, Yangzhou, Hunan, China; c Department of Orthopedics, Dalian Medical University, Dalian, Liaoning, China

**Keywords:** Osteosarcoma, bioinformatics, differentially expressed genes, functional enrichment analysis, protein–protein interaction

## Abstract

Osteosarcoma (OS) is the most common primary malignant tumor in children and adolescents. Numerous studies have reported the importance of miRNA in OS. The purpose of this study is to predict potential biomarkers and new therapeutic targets for OS diagnosis and prognosis by analyzing miRNAs of OS plasma samples from the Gene Expression Omnibus (GEO) database.

Data-sets were downloaded from the GEO and analyzed using R software. Different expressions of miRNAs (DE-miRNAs) in plasma and mRNAs (DE-mRNAs) in OS patients were identified. Funrich was used to predict the transcription factors and target genes of miRNAs. By comparing the target mRNAs and DE-mRNAs, the intersection mRNAs were identified. The intersection mRNAs were imported to perform Gene Ontology (GO) functional annotation and Kyoto Encyclopedia of Genes and Genomes (KEGG) pathway enrichment analysis. MiRNA-mRNA regulatory network and a protein-protein interaction (PPI) network were constructed by using Cytoscape. Finally, a total of 164 DE-miRNAs, 256 DE-mRNAs, and 76 intersection mRNAs were identified. The top 10 TF of up- and down-regulated DE-miRNAs were also predicted. In addition, GO and KEGG analyses further revealed the intersection mRNAs. By constructing the miRNA–mRNA networks, we found miR-30d-5p, miR-17-5p, miR-98-5p, miR-301a-3p, and miR-30e-5p were the central hubs. COL1A1, COL1A2, MMP2, CDH11, COL4A1 etc. were predicted to be the key mRNA by constructing the PPI networks. Through a comprehensive bioinformatics analysis of miRNAs and mRNAs in OS, we explored the potential effective biomarkers and novel therapeutic targets for the diagnosis and prognosis of OS.

## Graphical abstract

**Figure:**
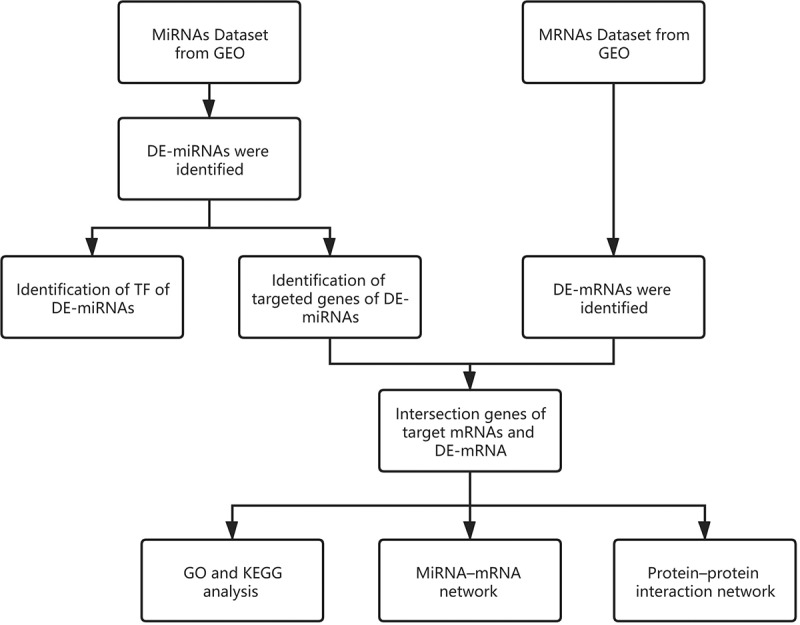

## Introduction

Osteosarcoma (OS) is the most common primary malignant bone tumor in children and adolescents, which has high mortality and disability rate [1–3]. With the progression of surgery, neoadjuvant chemotherapy, etc., the 5-year survival rate of patients with localized osteosarcoma has increased from less than 20% to 70%-80% [2,4]. However, the prognosis is still poor for patients with lung metastasis or recurrence. The 5-year survival rate of these patients is only 20% [4,5]. Therefore, it is necessary to further explore its potential pathogenesis.

MicroRNAs (miRNAs) are a class of non-coding small RNA about 20–24 nucleotides [6,7]. By targeting mRNA, miRNAs could regulate the process of biological processes, cell proliferation, cell division, apoptosis, cell invasion, metastasis, metabolism, and tumorigenesis [1,8,9]. Numerous studies have reported that abnormal expression of miRNAs is related to OS development and metastasis [8]. In addition, abnormal expressions of miR-21, miR-143, miR-145, miR-199a-3p, and miR-221 were found in the peripheral blood of OS patients [10–12].

Bioinformatics is a new field of biological research, processing, and analysis of biological data by using mathematical, statistical, and computational methods. Due to the massive amounts of data generated by new technologies, such as genomic sequencing and microarray chips, the traditional gene-by-gene approaches are insufficient to meet the growth and demand of biological research. Therefore, bioinformatics is a valuable way to achieve biological understanding and therapeutic progress [13].

MiRNAs play an important role in OS development and metastasis. Therefore, the aim of this study was to evaluate and summarize the evidence relating to the miRNA in OS plasma to determine the most effective diagnosis, treatment, and prognosis evaluation strategy.

## Materials & Methods

### Data processing of DE-miRNAs

We selected datasets from GEO (http://www.ncbi.nlm.nih.gov/geo), which is a publicly available database of gene and microarray profiles. The search strategy (‘osteosarcoma’ [MeSH Terms] and miRNA [All Fields]) AND (‘Homo sapiens’[Organism] AND ‘Expression profiling by RT-PCR’[Filter]) were adopted until August 2020. Inclusion criteria were as follows: plasma of miRNA from OS patients or healthy people. In the end, the data-set GSE65071 based on the platform of GPL19631 (Exiqon human V3 microRNA PCR panel I+ II) was chosen for analysis. Background corrections and normalizations were processed using the R package ‘affy’ from the Bioconductor project. The DE-miRNAs analysis was conducted using the R package ‘limma’. The P value <0.05 and |log2FC| >1 was set as the threshold for DE-miRNAs.

### Identification of TF and targeted genes of DE-miRNAs

The FunRich (http://www.funrich.org) provides annotations on pathways, TF, biological processes (BP), cellular components (CC), molecular functions (MF), etc. [14]. In the current study, up-regulated and down-regulated miRNAs were separately subjected to FunRich, top 10 TF of DE-miRNAs and targeted genes were separately identified.

### Data processing of DE-mRNAs

We selected GEO and the search strategy (‘osteosarcoma’ [MeSH Terms] and mRNA [All Fields]) AND (‘Homo sapiens’[Organism] AND ‘Expression profiling by array’[Filter]) were adopted until August 2020. Inclusion criteria were as follows: mRNAs from patients with OS or healthy people. In the end, the data-set GSE16088 based on the platform of GPL96 (Affymetrix Human Genome U133A Array) was chosen for analysis. Background correction, normalization and log2 transformation were processed by the R package ‘affy’ from the Bioconductor project. The differential analysis was conducted using the R package ‘limma’. The P value <0.05 and |log2FC| >2 was set as the criterion for DE-mRNAs.

### Intersection genes of target mRNAs and DE-mRNAs

The target genes of DE-miRNAs predicted by FunRich were compared with DE-mRNAs predicted by GEO. The intersection genes were presented as Venn diagrams and the overlap mRNAs were identified. It is widely acknowledged that there is an inverse relationship between miRNA and the expression of target genes. As a result, upregulated mRNA and downregulated mRNA were defined.

### Functional annotation and pathway enrichment analysis

GO functional annotation is a widely used bioinformatics tool for analyzing functional relationships between gene products, including three categories: CC, BP, and MF. KEGG analysis is used to study the enrichment pathway of cross genes, so as to further understand gene function. GO functional annotation and KEGG pathway enrichment analysis were performed by the R package ‘cluster Profiler’ and ‘enrich plot’ [14]. Fisher’s exact test was used to classify the GO category and select the significant KEGG pathway, P value < 0.05 and q value < 0.05 were considered a significant difference.

### Construction of miRNA–mRNA network and Protein–protein interaction network

The miRNA–mRNA network of the mRNAs was constructed by Cytoscape v3.8.0 [15,16]. The PPI network for dysregulated mRNAs was established via the Search Tool for the Retrieval of Interacting Genes database (STRING, http://string-db.org). Then, the PPI network was constructed by Cytoscape. Based on the combined score>0.4 for PPI pairs of DEGs in our study, we further built a PPI network model using Cytoscape software.

### Identification of potential Hub mRNAs and hub miRNAs

We calculated the node number of each miRNA to identify hub regulators in the above miRNA-mRNA network. MiRNAs with more than 5 nodes in the network were selected as hub-miRNAs. Higher degree nodes play a vital role in maintaining the stability of the whole network.

## Results

The goal of this study was to evaluate and summarize the evidence relating to the miRNA in OS plasma to determine the most effective diagnosis, treatment, and prognosis evaluation strategy. We predicted TF of miRNAs, pathways hub miRNAs, and hub mRNAs and Constructed of miRNA–mRNA network and PPI network.

## Identification of DE-miRNAs and DE-mRNAs

**Figure 1.**
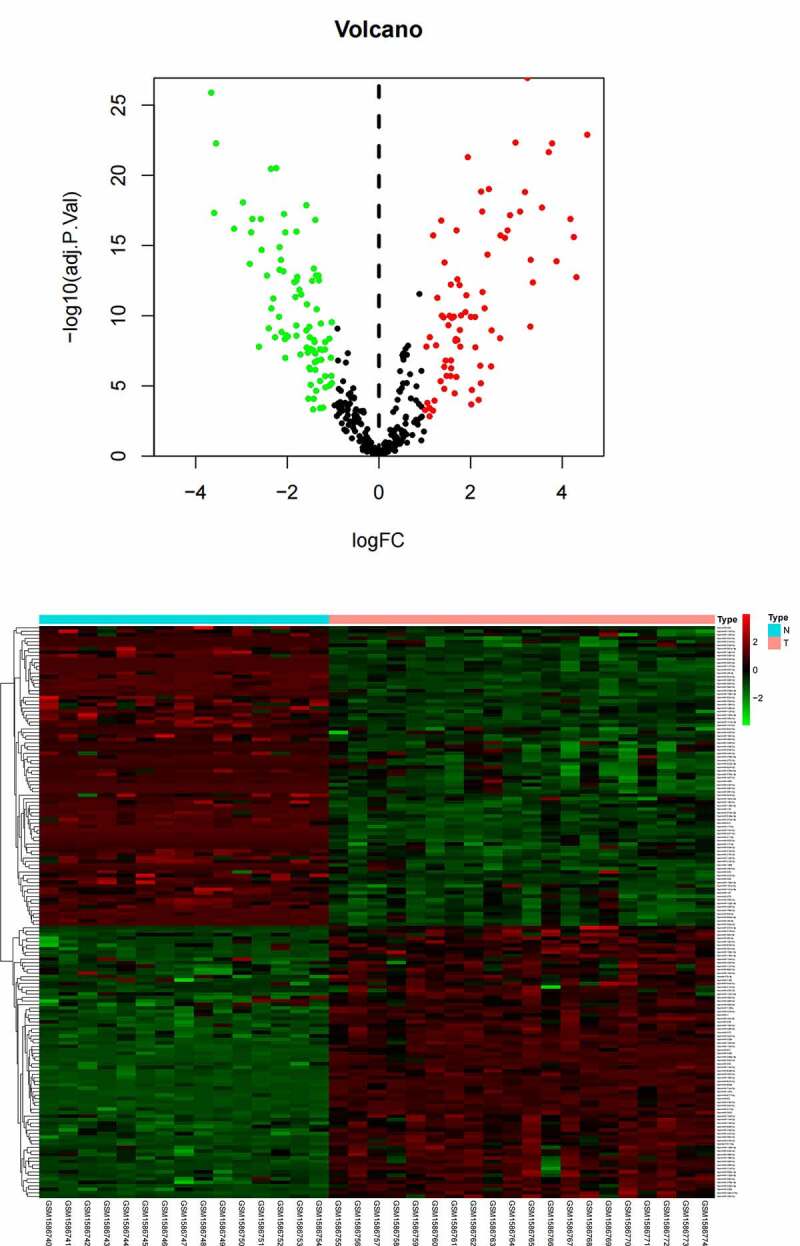
(a) Volcano plots of DE-miRNAs. (b) Heatmap of DE-miRNAs

**Figure 2.**
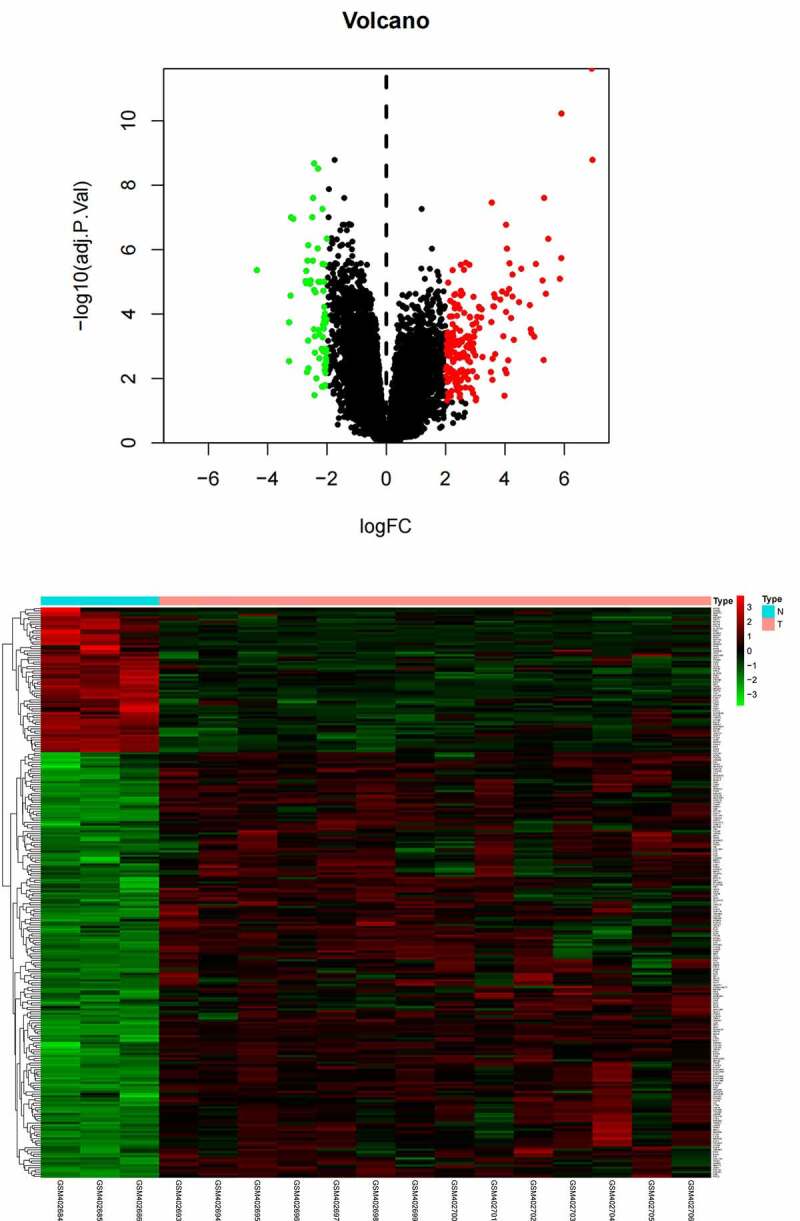
(a) Volcano plots of DE-mRNAs. (b) Heatmap of DE-mRNAs

A total of 164 DE-miRNAs from the plasma samples (78 up- and 86 down-regulated DE-miRNAs) were identified between 15 healthy people and 20 OS patients. Volcano plot (Figure 1a) and Heatmap (Figure 1b) were established to plot the DE-miRNAs. The DE-mRNAs of 14 OS patients and 3 healthy controls were analyzed, and a total of 256 DE-mRNAs (191 up- and 65 down-regulated DE-mRNAs) were identified. Volcano plot (Figure 2a) and Heatmap (Figure 2b) were established to plot the DE-mRNAs. Top 20 differentially expressed miRNAs and mRNAs were shown in Table 1A and 1B.

**Table 1A.** The table shows the information of Top 20 differentially expressed miRNAs

ID	logFC	adj.P.Val	Regulated
hsa-miR-671-5p	4.546784	1.29E-23	up
hsa-miR-375	4.310829	1.81E-13	up
hsa-miR-490-3p	4.253599	2.50E-16	up
hsa-miR-203a	4.17923	1.29E-17	up
hsa-miR-150-5p	3.876575	1.32E-14	up
hsa-miR-499a-5p	3.780643	5.35E-23	up
hsa-miR-663a	3.706472	2.24E-22	up
hsa-miR-744-5p	−3.6605 C9	1.29E-26	down
hsa-miR-127-3p	−3.59745	4.82E-18	down
hsa-miR-31-5p	3.558546	1.98E-18	up
hsa-miR-331-3p	−3.55621	5.35E-23	down
hsa-miR-206	3.360764	4.28E-13	up
hsa-miR-144-5p	3.312609	1.06E-14	up
hsa-miR-10b-5p	3.305259	5.98E-10	up
hsa-miR-624-5p	3.2403	1.18E-27	up
hsa-miR-181d	3.186622	1.56E-19	up
hsa-miR-503-5p	−3.16267	6.39E-17	down
hsa-miR-502-5p	3.080622	3.82E-18	up
hsa-miR-95	2.981734	4.65E-23	up
hsa-miR-337-3p	−2.96696	8.44E-19	down

**Table 1B.** The table shows the information of Top 20 differentially expressed mRNAs

ID	logFC	adj.P.Val	Regulated
SPP1	6.949825862	1.64E-09	up
SEPP1	6.923031057	2.41E-12	up
MAFB	5.90485027	5.97E-11	up
LUM	5.900095106	1.84E-06	up
CTSK	5.850469544	8.03E-06	up
A2M	5.45878503	4.63E-07	up
COL1A2	5.379133987	2.35E-05	up
OLFML2B	5.320771951	2.49E-08	up
IBSP	5.302998023	0.002672362	up
ENPP2	5.265582743	9.05E-06	up
HLA-DRA	5.040214057	2.79E-06	up
MMP9	4.989747551	0.000505257	up
HBB	4.891442564	0.000388009	up
MGP	4.868326448	0.000297807	up
LYZ	4.836968258	5.28E-05	up
COL15A1	4.548971176	3.93E-06	up
MXRA5	4.474484032	4.27E-05	up
KRT18	−4.363851615	4.35E-06	down
DCN	4.299072964	0.000630991	up
LRRC15	4.253669709	5.95E-06	up

## Predicting TF and target genes of miRNAs

The top 10 TF of up-regulated DE-miRNAs (Figure 3a) were EGR1, SP1, SP4, POU2F1, MEF2A, NKX6-1, NFIC, RREB1, ZFP161, FOXA1. The top 10 TF of down-regulated DE-miRNAs (Figure 3b) were EGR1, SP1, POU2F1, SP4, FOXA1, MEF2A, ZFP161, NFIC, NKX6-1, LHX3. The target mRNAs of 78 up- and 86 down-regulated DE-miRNAs were successfully predicted, 4638 up- and 6069 down-regulated target mRNAs were identified.

**Figure 3.**
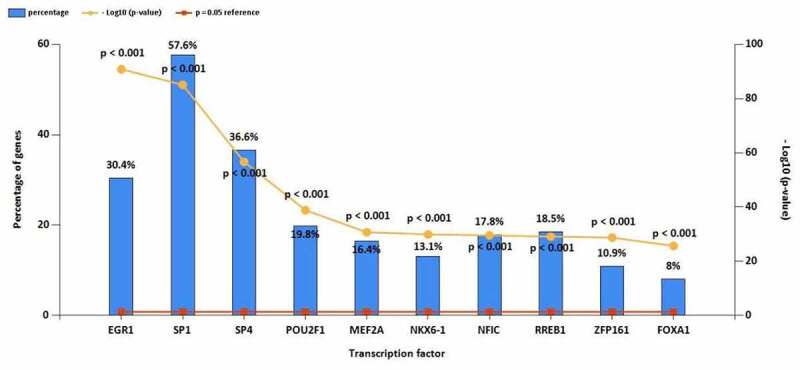
(a) Top 10 TF of up-regulated DE-miRNAs. (b) Top 10 TF of down-regulated DE-miRNAs

**Figure 3.**
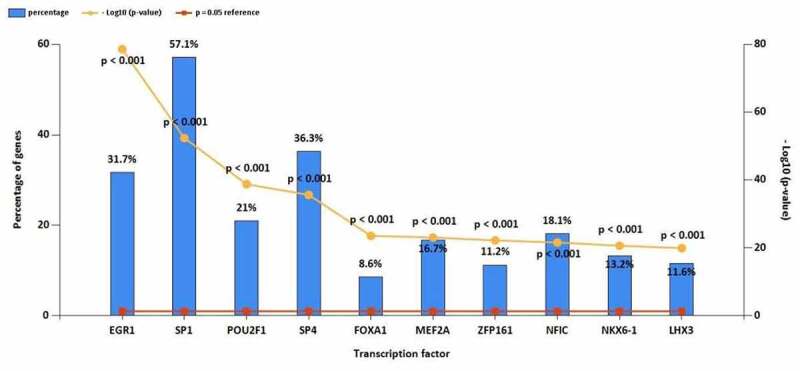
Continued


## Identifying the intersection mRNAs

**Figure 4.**
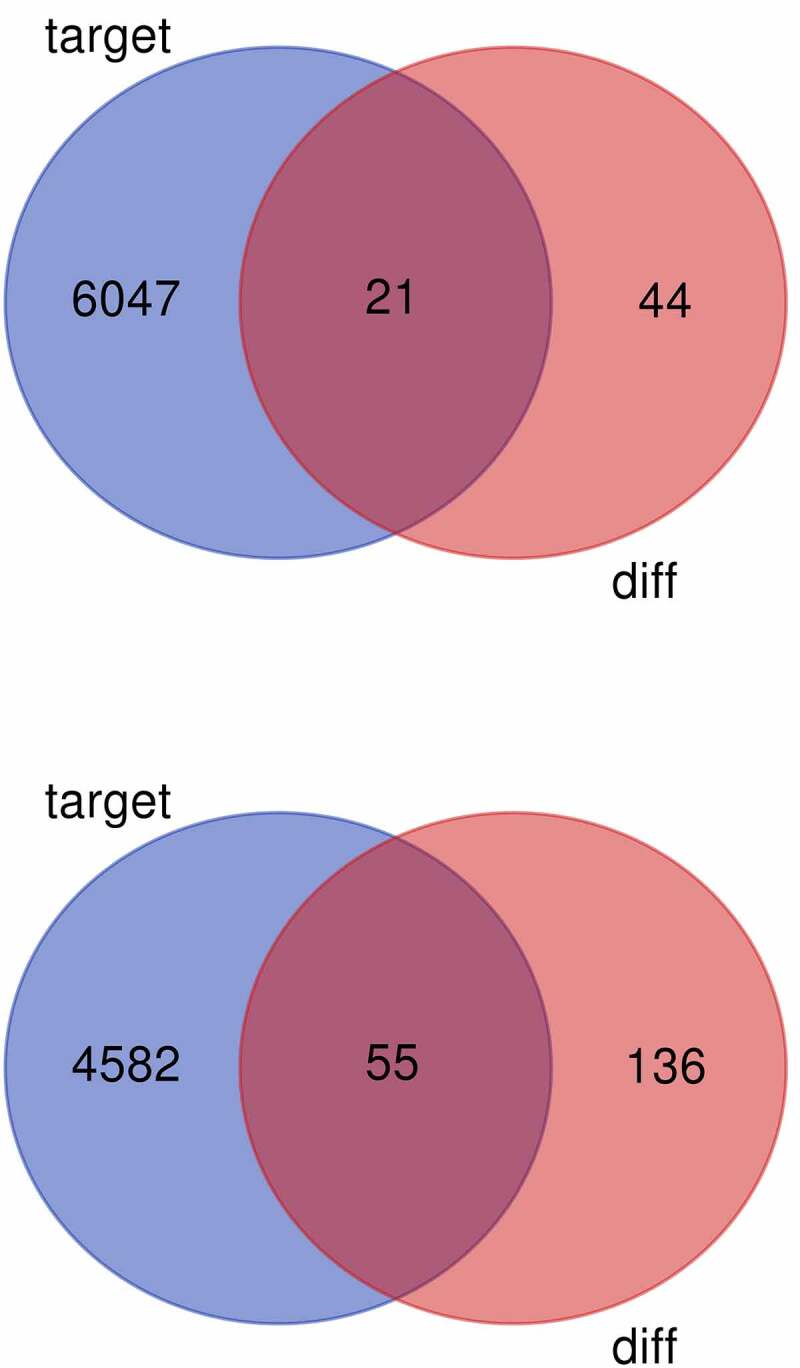
(a) Venn diagram of common differentially expressed genes from the two datasets. DE-mRNAs were down-regulated in the dataset. (b) Venn diagram of common differentially expressed genes from the two datasets. DE-mRNAs were up-regulated in the dataset

A total of 6069 target genes predicted from up-regulated DE-miRNAs were compared with 65 down-regulated DE-mRNAs, the overlap 21 mRNAs were presented as Venn diagram (Figure 4a). A total of 4638 target genes predicted from down-regulated DE-miRNAs were compared with 191 up-regulated DE-mRNAs, the overlap 55 mRNAs were presented as Venn diagram (Figure 4b). The intersection genes were shown in Table 2.

**Table 2.** The table shows the information of intersection genes

mRNA	Genes name
Upregulated	MMP2, COL1A1, TMEM59, OPN3, CDH11, DAAM2, PABPN1, VCAN, SULF1, MYO1D, TWIST1, COL1A2, SAT1, MEF2C, PTN, GPR137B, YTHDF3, SATB2, TMEM47, OXR1, DDIT4, FOS, PTBP2, DDR2, COL5A1, GHR, EVI2A, NDNF, PRRX1, SPP1, CTSK, CSGALNACT1, NID2, COL16A1, MAFB, P4HA1, COL4A1, ATP10D, PLEKHA5, SATB1, VAMP8, GJA1, BTG1, COL15A1, LRRC17, MARCKS, FAP, FAM46A, CSAD, DLX5, ZEB2, OLFML2B, LPAR6, LRP4, SNAI2
Downregulated	ITGA3, GCLM, SNAPC1, PPID, OIP5, KIF23, TUBB2B, PAX6, ODC1, FZD2, CTGF, ZNF580, TFDP1, DRAP1, FOSL1, TLN1, S100A2, TPM2, FHL2, KHDRBS3, HMGA1

## Functional annotation and pathway enrichment analysis

GO (Figure 5a, Figure 5b) and KEGG pathway (Figure 5c, Figure 5d) enrichment analysis were performed to understand the biological meanings of the 76 dysregulated mRNAs. BP terms of 76 dysregulated mRNAs were significantly enriched in extracellular matrix organization, ossification, osteoblast differentiation, and etc. CC terms of the genes were mostly enriched in collagen−containing extracellular matrix, focal adhesion, cell−substrate junction, and etc. MF terms of the genes were mostly enriched in DNA−binding transcription activator activity, RNA polymerase II−specific, DNA−binding transcription activator activity, extracellular matrix structural constituent, integrin binding, and et al. KEGG analysis indicated that these genes were mainly involved in ECM−receptor interaction, Protein digestion and absorption, PI3K−Akt signaling pathway, Relaxin signaling pathway, Focal adhesion, and etc.

Besides, 55 up-regulated mRNAs were present to perform GO (Figure 6a, Figure 6b) and KEGG pathway (Figure 6c, Figure 6d) enrichment analysis. 21 down-regulated mRNAs were also present to perform GO (Figure 7a, Figure 7b) and KEGG pathway enrichment analysis. But this list of down-regulated mRNAs had no outcome of the KEGG pathway.

**Figure 5.**
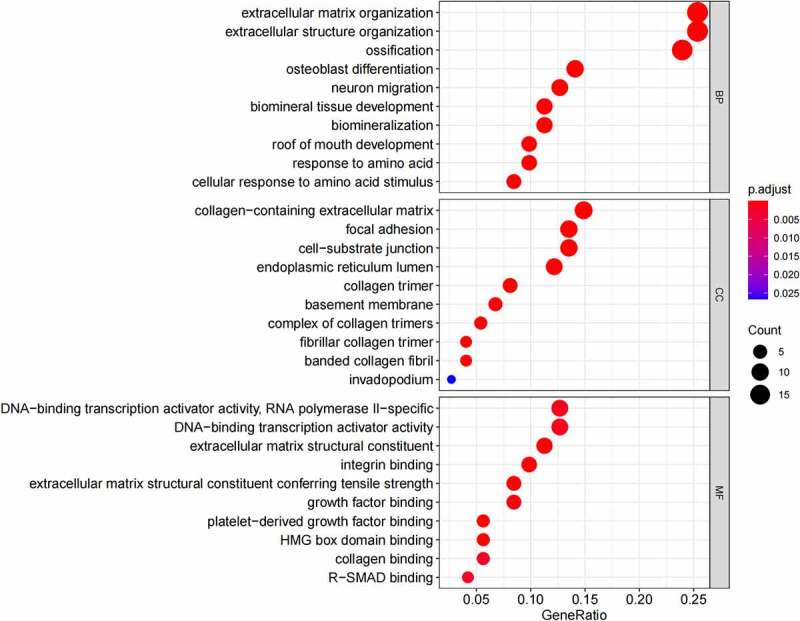
(a) GO functional annotation of 76 dysregulated mRNAs. Top 10 GO terms of 76 dysregulated mRNAs in three categories (CC, BP, MF). (b) GO functional annotation of 76 dysregulated mRNAs. Top GO terms and their enriched mRNAs. (c) KEGG pathway enrichment analysis of 76 dysregulated mRNAs. Top 10 KEGG enrichment pathways of 76 dysregulated mRNAs. (d) KEGG pathway enrichment analysis of 76 dysregulated mRNAs. Top 10 KEGG enrichment pathways and their enriched mRNAs

**Figure 5.**
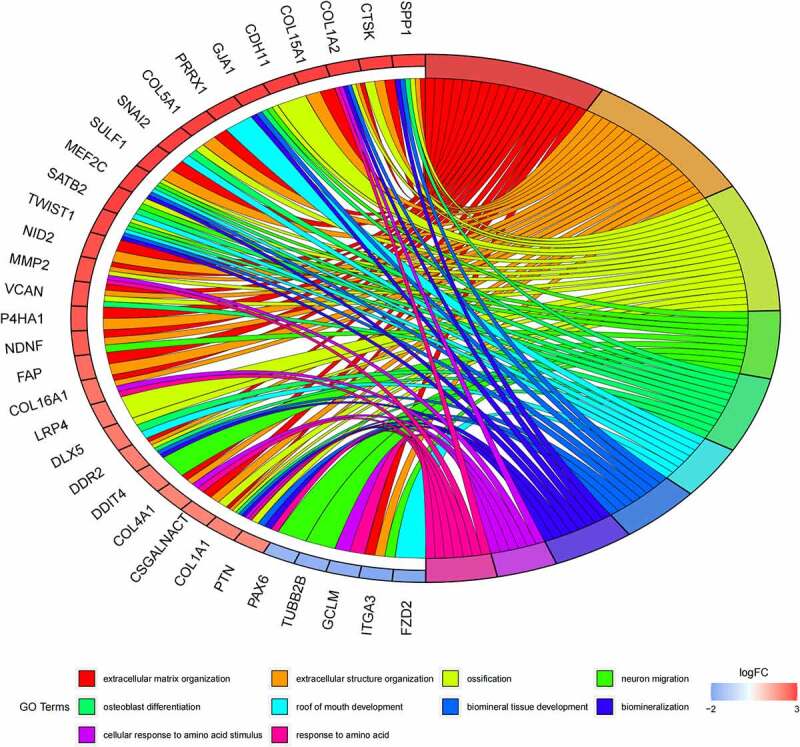
Continued

**Figure 5.**
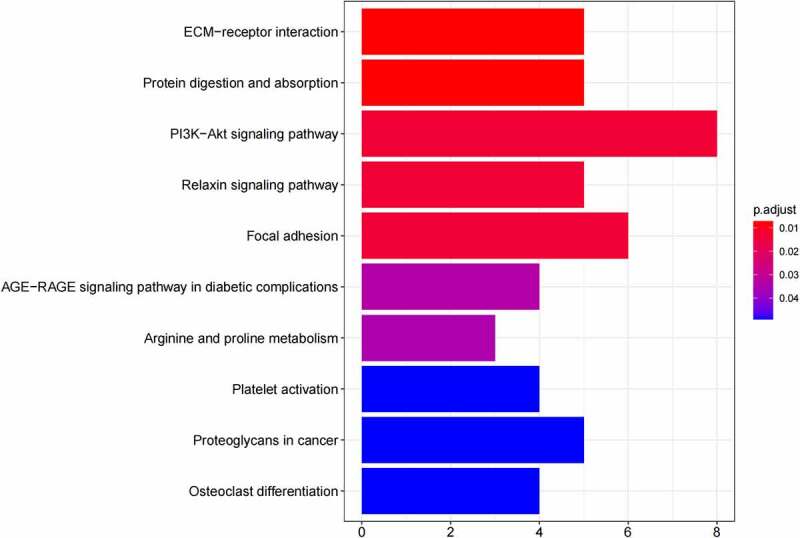
Continued

**Figure 5.**
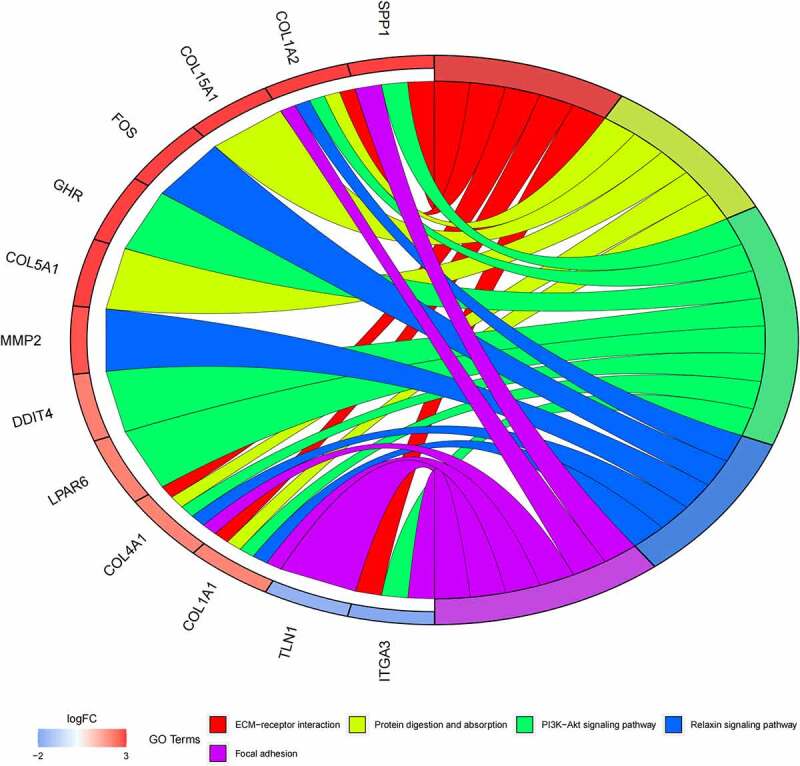
Continued
**Figure 6.**
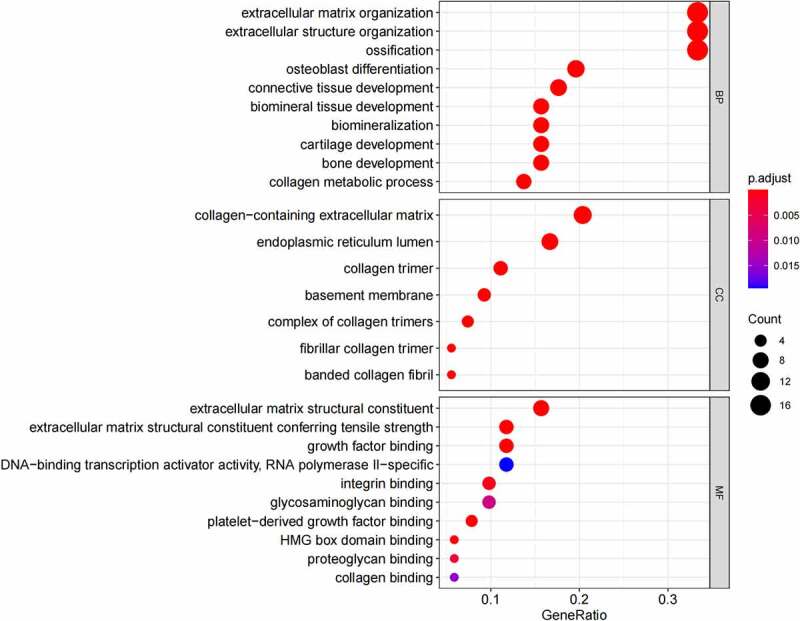
(a) GO functional annotation of 55 up-regulated mRNAs. Top 10 GO terms of 55 up-regulated mRNAs in three categories (CC, BP, MF). (b) GO functional annotation of 55 up-regulated mRNAs. Top GO terms and their enriched mRNAs. (c) KEGG pathway enrichment analysis of 55 up-regulated. Top 10 KEGG enrichment pathways of 55 up-regulated mRNAs. (d) KEGG pathway enrichment analysis of 55 up-regulated mRNAs. Top 10 KEGG enrichment pathways and their enriched mRNAs

**Figure 6.**
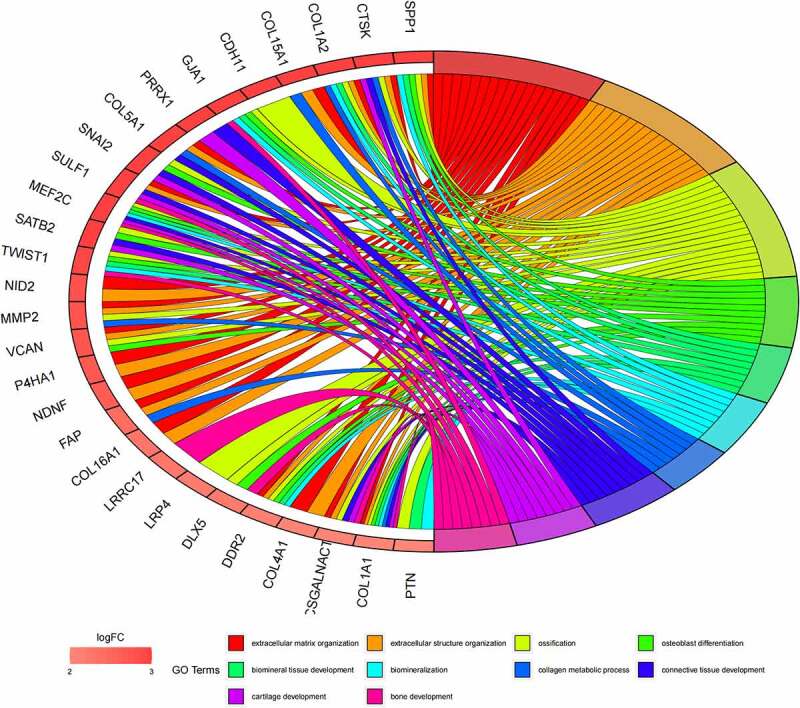
Continued

**Figure 6.**
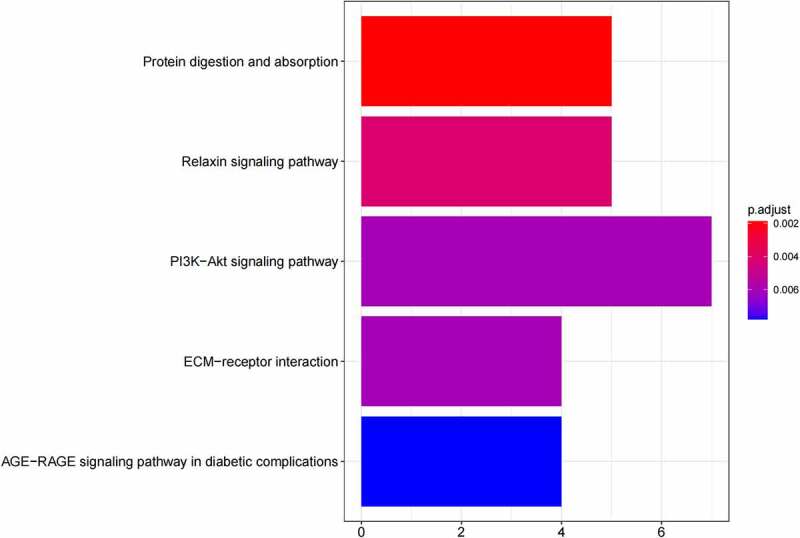
Continued

**Figure 6.**
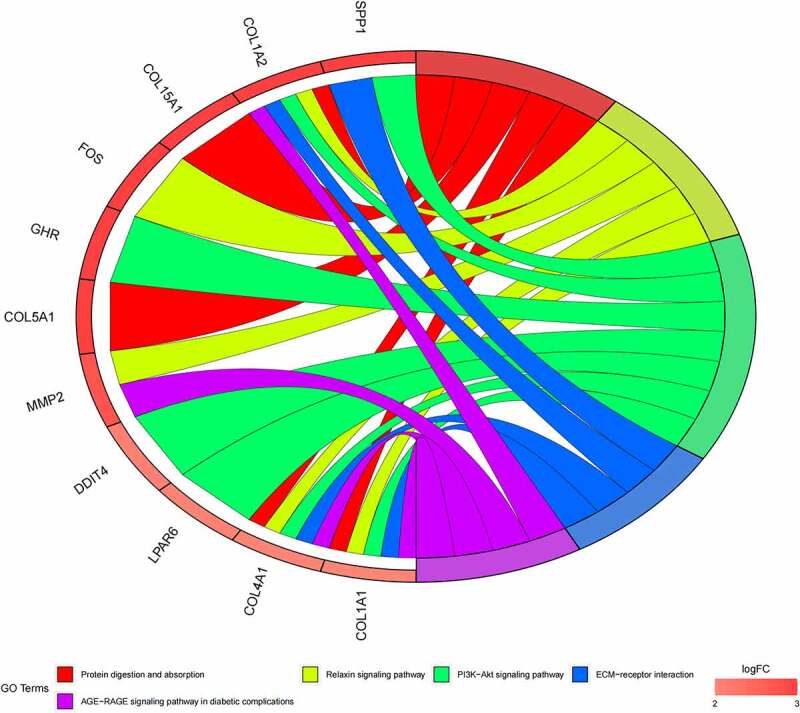
Continued

**Figure 7.**
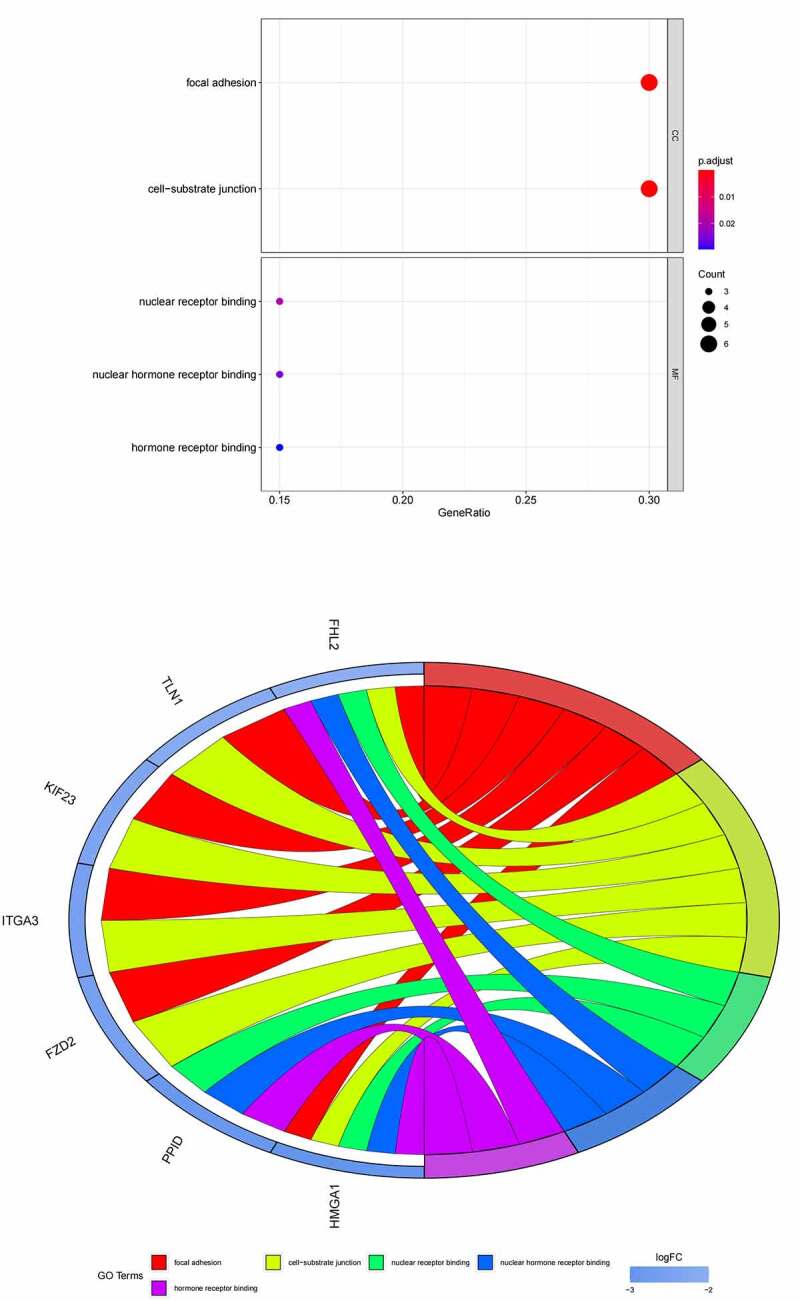
(a) GO functional annotation of 21 down-regulated mRNAs. Top 10 GO terms of 21 down-regulated mRNAs in three categories (CC, BP, MF). (b) GO functional annotation of 21 down-regulated mRNAs. Top GO terms and their enriched mRNAs

## Construction of miRNA–mRNA network and PPI network

**Figure 8.**
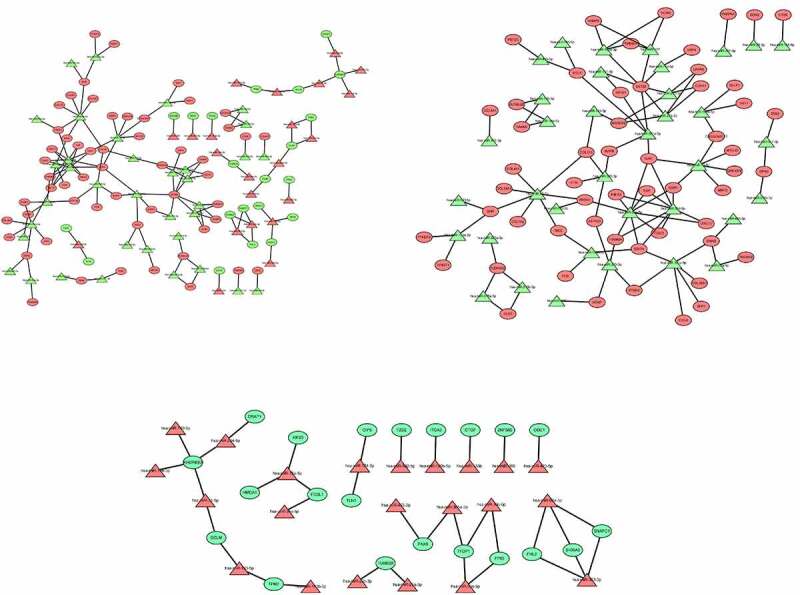
(a) The miRNA–mRNA networks of all dysregulated mRNAs. (b) The miRNA–mRNA networks of up-regulated mRNAs. (c) The miRNA–mRNA networks of down-regulated mRNAs

**Figure 9.**
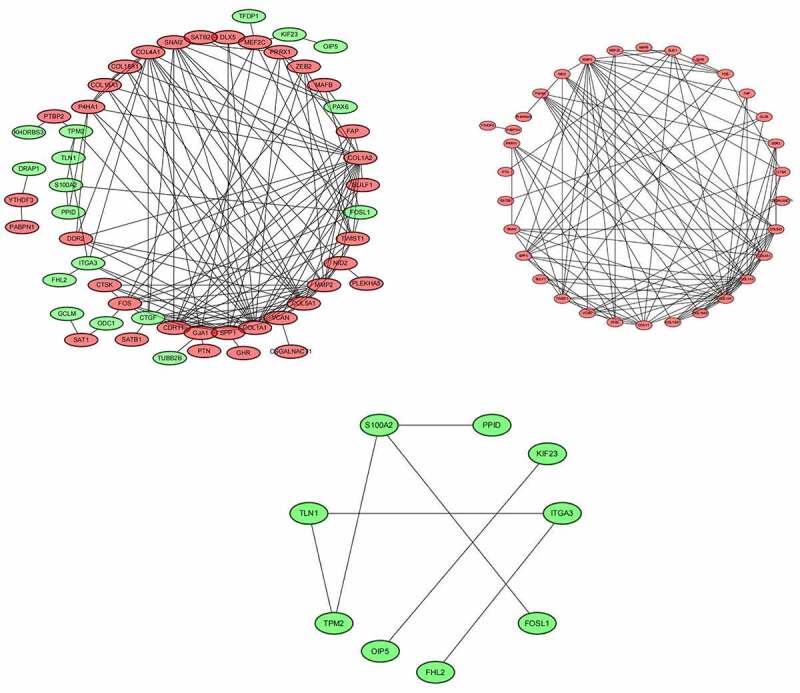
(a) PPI network of all dysregulated mRNAs. (b) PPI network of up-regulated mRNAs. (c) PPI network of down-regulated mRNAs

The miRNA–mRNA networks of all dysregulated mRNAs (Figure 8a) up-regulated mRNAs (Figure 8b), and down-regulated mRNAs (Figure 8c) were constructed by Cytoscape software. PPI network of all dysregulated mRNAs (Figure 9a), up-regulated mRNAs (Figure 9b), and down-regulated mRNAs (Figure 9c) were constructed using the STRING database and Cytoscape software.

## Hub mRNAs and hub miRNAs

MiR-30d-5p, miR-17-5p, miR-98-5p, miR-301a-3p, and miR-30e-5p were selected as hub-miRNAs, which reflected their major role in the regulation network. The information of hub miRNAs was shown in Table 3. The top 20 hub mRNAs were COL1A1, COL1A2, MMP2, CDH11, COL4A1, COL5A1, CTGF, SPP1, TWIST1, FOS, GJA1, SNAI2, P4HA1, VCAN, MEF2C, NID2, ZEB2, COL15A1, COL16A1, and DDR2. The information of hub mRNAs was shown in Table 4.

**Table 3.** The table shows the information of hub miRNAs

miRNA	Type	mRNA
hsa-miR-30d-5p	Down	CSAD, DDIT4, FAM46A, FAP, GJA1, LRRC17, OXR1, P4HA1
hsa-miR-17-5p	Down	CSGALNACT1, GJA1, GPR137B, MMP2, MYO1D, OXR1
hsa-miR-98-5p	Down	COL15A1,COL1A1, COL1A2, COL4A1, GHR, NID2, PRRX1
hsa-miR-301a-3p	Down	BTG1, GJA1, MAFB, SATB1, SATB2
hsa-miR-30e-5p	Down	CSAD, DDIT4, FAM46A, FAP, GJA1, LRRC17, OXR1, P4HA1, PRRX1
hsa-miR-181 c-5p	Down	COL16A1, DDIT4, EVI2A, PTBP2, SNAI2, SPP1

**Table 4.** The table shows the information of hub mRNAs, the information of hub mRNAs

mRNAs	Type	Degree	mRNAs	Type	Degree
COL1A1	Up	23	GJA1	Up	10
COL1A2	Up	20	SNAI2	Up	10
MMP2	Up	17	P4HA1	Up	8
CDH11	Up	13	VCAN	Up	8
COL4A1	Up	13	MEF2C	Up	7
COL5A1	Up	13	NID2	Up	7
CTGF	Down	12	ZEB2	Up	7
SPP1	Up	12	COL15A1	Up	6
TWIST1	Up	11	COL16A1	Up	6
FOS	Up	10	DDR2	Up	6

## Discussion

Previous studies have reported that miRNAs play an important role in the occurrence and development of OS, but the interaction between miRNAs and mRNA is still unclear [5]. The miRNA-mRNA regulatory network plays an important role in the occurrence and development of cancer.

Based on the GSE65071 profile dataset, 15 primary normal plasmas, 20 OS plasmas, were enrolled and analyzed. A total of 256 DE-miRNAs (191 up- and 65 down-regulated DE-miRNAs) were identified. Five miRNAs including miR-30d-5p, miR-17-5p, miR-98-5p, miR-301a-3p, and miR-30e-5p were identified as the hub miRNAs in our network. Liao et al. found miR-98-5p was sponged with LncRNA SNHG16 to regulate cellular processes in OS [17]. Many studies have reported that microRNA-301a promoted cell proliferation in OS and other sarcomas [18–21]. The normal expression of miR-30d-5p could inhibit cancer proliferation, migration, and invasion of non-small cell lung cancer [22]. It was reported that a low expression of miR-30e-5p was associated with many kinds of cancers, such as nasopharyngeal carcinoma, esophageal cancer, and bladder cancer [8,23,24]. However, a previous study reported miR-17-5p is down-regulated in the OS patients, which is contrary to our results [7]. It may be related to different types of specimens.

TFs and miRNAs regulate mRNA gene expression. Additionally, miRNAs and TF could alter the expression of each other [25]. We predicted the TF of DE-miRNA using FunRich software such as EGR1, Sp1, SP4, POU2F1. Early growth response protein-1 (EGR1) is a zinc-finger protein, belonging to the EGR family [9]. Cell differentiation and mitogenesis require the participation of target gene products activated by the EGR family [26]. EGR1 is reported to be a cancer suppressor gene and the downregulating of EGR1 in OS samples was usually associated with poor prognosis [9]. Fiorillo et al. found EGR1 could regulate MG-specific miRNAs, miR-21-5p and miR-30e-5p [27]. Sp1 is related to many cellular processes, including cell growth, cell differentiation, immune responses, apoptosis, chromatin remodeling, and response to DNA damage [28–30]. Xing et al. reported that SP1 promoted OS progression via the miR-655/SOX18 axis [28]. Gao et al. found that Sp1-mediated up-regulation of lnc00152 promotes invasion and metastasis of retinoblastoma cells via the miR-30d/SOX9/ZEB2 pathway [31]. POU2F1 is also known as OCT-1, located on chromosome 1q24 [32]. The POU2F1 transcription factor belongs to the POU transcription factor family that contains the POU domain with a necessary amino acid region for DNA binding to the octameric sequence ATGCAAAT [33]. Li et al. reported that LncRNA SND1-IT1 via sponging miRNA-665 upregulated POU2F1 to accelerate the proliferation and migration of OS [32].

Using target genes for DE-miRNAs overlapped with DE-mRNAs from GSE16088 profile dataset, 55 up-, and 21 downregulated target mRNAs were identified. According to GO analysis, the 76 mRNAs enriched in osteoblast differentiation, focal adhesion, and integrin binding. Bone remodeling is an important process, which is the balancing activities of the bone-forming osteoblasts and the bone-resorbing osteoclasts [34]. Impairments in these balanced activities may result in osteo-condensing bone pathologies, such as osteoporosis, osteopetrosis, and can also be the origin of bone cancers. The suspected initiated cell in OS is the osteoblast [35]. Tanaka et al. reported that miR-138 could inhibit Ewing’s sarcoma cells metastatically by targeting focal adhesion kinase [36]. Liu et al. found that miR-128 inhibited epithelial–mesenchymal transition of human OS cells by directly targeting integrin α2 [37]. KEGG analysis found that these genes were mainly involved in ECM−receptor interaction, protein digestion, and absorption, PI3K−Akt signaling pathway, Relaxin signaling pathway, and Focal adhesion. The occurrence, development, invasion, and metastasis of malignant tumors are often related to the abnormal expression of ECM and ECM−receptor [38,39]. The tumor cells activate or secrete protein-degrading enzymes to degrade the matrix after adhering to various components of the ECM through their surface receptors, thereby forming a local lysis zone, which constitutes a channel for tumor cell metastasis. Chen et al. reported miR-191-5p promoted the development of OS via targeting EGR1 and activating the PI3K/AKT signaling pathway [9]. These different pathways may be the potential mechanism of OS.

In order to construct a PPI network of target genes, we screened out the top 20 genes. There were COL1A1, COL1A2, MMP2, CDH11, etc. COL1A1 has a triple helix structure, comprising two alpha1 chains and one alpha2 chain, which encodes the pro-alpha1 chains of type I collagen. It is associated with a particular type of skin tumor, and is promoted through overproduction of platelet-derived growth factor (PDGF), such as dermatofibrosarcoma [40]. Bi et al. reported that MicroRNA-98 inhibited the cell proliferation of human hypertrophic scar fibroblasts by targeting Col1A1 [41]. In clinical melanoma samples, the upregulation of COL1A1 was negatively correlated with disease-free survival [42]. MMP2 is a member of the MMP gene family, which has the ability to cleave ECM components and participate in signal transduction. MMPs and their endogenous inhibitors (tissue inhibitors of metalloproteinases, TIMPs) are the most important substances in the regulation of the ECM degradation process [39]. The imbalance of MMP-TIMP results in associating tumor invasion by extracellular matrix proteolysis. The previous study has reported overexpression of MMP-2 and MMP-3 promoted OS cell migration [43]. Gong et al. found that up-regulated miR-17 in ovarian cancer cells could decrease the production of activated MMP-2 and inhibit ovarian cancer cell peritoneal metastasis [44].In addition, Jia et al. reported downregulation of TWIST1 decreased cell viability, inhibited migration, and promoted apoptosis of OS cells [45], Sun et al. found miR-143-3p inhibited the proliferation, migration, and invasion of OS cells by targeting FOSL2 [46]. Croset et almiR-30 could inhabit tumor cell invasion by silencing CDH11 or ITGA5 in ER-/PR-negative breast cancer [47]. By analyzing the miRNA of the blood of patients with OS, we can predict potentially effective biomarkers associated with the invasion and metastasis of OS, which provides a new perspective on early diagnosis and finding novel therapeutic targets for OS patients. However, the present study has several limitations. First of all, this study lacks more convincing evidence such as qPCR and immunohistochemistry results. Secondly, there are only sarcoma data in the gepia database, while there are only two normal samples in the normal group so we cannot provide the expression level of hub genes between OS and normal samples in the public database. Third, the present data was obtained from the GEO database, which cannot evaluate the reliability and quality of statistical data. Fourth, this study lacks prognosis information from public databases.

## Conclusions

Through a comprehensive bioinformatics analysis of miRNAs and mRNAs in OS, we explored the potential effective biomarkers and novel therapeutic targets for the diagnosis and prognosis of OS.

## Data Availability

All data are fully available without restriction.

## Highlights

1. **We predicted underlying TF and target genes of DE-miRNAs from OS plasma samples.**
2. **Based on the blood samples of OS patients, GO functional annotation and KEGG pathway enrichment analysis were performed.**
3. **Based on the blood samples of OS patients, MiRNA-mRNA network, and PPI network were constructed.**
